# Message Sidedness in Health Claims: Roles of Mood State, Product Involvement, and Self-Rated Health Status

**DOI:** 10.3389/fnut.2021.729370

**Published:** 2021-12-14

**Authors:** Hung-Chou Lin, Shih-Tse Wang

**Affiliations:** ^1^Department of Adult and Continuing Education, National Taiwan Normal University, Taipei City, Taiwan; ^2^Graduate Institute of Bio-Industry Management, National Chung Hsing University, Taichung, Taiwan

**Keywords:** message sidedness, heuristic-systematic model, food product evaluation, mood state, product involvement, self-rated health

## Abstract

Most of the previous studies with respect to message sidedness mainly focus on the effect of message sidedness in advertising on behavior of consumers and it is unknown how consumers respond to different message sidedness when a one-sided or two-sided message in claims shown on the package of a healthy food product. This study explores the underlying mechanisms how consumers respond to different message sidedness in claims. The results indicate that two-sided messages in claims are more persuasive than one-sided messages because they pass the “sufficiency threshold.” In addition, the results of this article show that mood state, product involvement, and self-rated health of individuals moderate the relationship between message sidedness in claims and product evaluation.

## Introduction

Food products not only provide necessary nutrition for humans, but also improve the physical and mental well-being of individuals (1, 2). Therefore, an increasing number of people believe that food products directly contribute to their health (3, 4). Consumers are becoming increasingly health conscious and are also willing to opt for health-promoting food products to transform their eating habits (5). A highly effective means of enabling people to make healthy food choices is providing labels and health claims on food products (6). Studies have also reported that health claims effectively influence health beliefs and purchase intentions of consumers (7, 8). Researchers have ascertained that consumers perceive a product labeled with health and nutrition claims to be healthier than a product without any claim [(8–10)]. In the global food and beverage market, health is considered the most crucial trend and innovation driver (11); therefore, an increasing number of food product companies are including health-related arguments into their communication strategies to appeal to health-conscious consumers.

The food industry prefers using health claims with positive contributions to life (life marketing) instead of those with negative influences on life (death marketing) (12). Van Kleef et al. (13) argued that the success of life or death marketing of a product depends on health claims made by the product. Levin et al. (14) proposed that advertising with negative information attracts more attention and in-depth information processing than advertising with positive information. In addition, Lin and Lin (15) demonstrated that people are more likely to focus on negative information than on positive information. However, life or death marketing is a more effective strategy that remains unclear. Moreover, most studies investigating the effects of health claims, either positive or negative, on product evaluation of consumers have included only supporting arguments and studies that also address opposing viewpoints are limited. Messages that contain only supporting arguments have been categorized as one-sided messages, whereas those messages that contain negative information in addition to supporting arguments have been categorized as two-sided messages (16, 17). O'Keefe (18) proposed the “message sidedness effect” that refers to the persuasive effect of either a one- or two-sided message on attitude or belief change. A one- or two-sided health claim can enhance the persuasiveness of communication that remains unclear (19). Therefore, this study explored whether, and under what conditions, a one- or two-sided health claim enhances the persuasive effects of healthy food product evaluation of consumers. The heuristic–systematic model (HSM) proposed by Chaiken (20) revealed that individuals with different motivation and ability have different information processing mode. The heuristic processing mode and the systematic processing mode from the HSM have been investigated in many studies (16, 21). This study utilized the HSM to better explain the effectiveness of one- and two-sided message.

This study focused on how consumers respond to different message sidedness when they choose a healthy food product. In addition, alternative moderators of message sidedness effects, such as mood states, product involvement, and self-perceived health status, were incorporated into the discussion to assess the differential persuasive effects of one- and two-sided messages.

The mood states of consumers play a major role in the manner in which they learn, remember, think, and evaluate complex social information (22–24). Consumers in different mood states are likely to perform different evaluations and exhibit different attitudes when presented with messages and information. Moreover, Köster and Mojet (25) proposed a bidirectional relationship between mood states and foods and argued that mood states affect food choice and food intake and food consumption, in turn, influences feelings of people. Hence, investigating how consumers in different mood states respond to message sidedness while searching for healthy food products is crucial (26).

Consumer product involvement has been reported to influence brand loyalty, responses to product information and advertising, and even purchase decisions of consumers (27, 28). Moreover, consumer involvement has been considered crucial for creating improved new products (29). Before making purchase decisions with respect to related products, consumers probably invest considerable amounts of time and effort in obtaining relevant information. Investigating the role of consumer involvement in product evaluation is necessary when consumers process messages with different sidedness are warranted.

Keller et al. (30) indicated that reading and processing health claims should be considered while assessing whether motivation of consumers has an influence on their perception of the health benefits of products. Consumers have been found to perceive health claims differently after a change in their health status. Investigating the role of self-rated health in personal relevance and the response to healthy food products is warranted. Therefore, this study investigated how consumers respond to messages with different sidedness while choosing healthy food products and the moderating roles of mood states, product involvement, and self-rated health status and their effects on the relationship between message sidedness and healthy food product evaluation of consumers.

## Literature Review

### Effects of Message Sidedness on Evaluation of Healthy Food Products of Consumers

Marketers who design health claims typically present their products to consumers in a favorable manner. For example, the purpose of marketing strategies (life or death marketing) is to persuade and convince consumers by presenting positive product information (e.g., claims of enhanced function and reduced disease risk). However, marketing studies have suggested that product-related messages that include some negative information are still more effective than those that include no negative information, especially in terms of enhancing credibility. However, studies on two-sided advertising have reported ambiguous and equivocal findings and indicated a complex relationship between message sidedness and communication effects in marketing. For example, relevant studies have demonstrated that a two-sided message is likely to inhibit consumers from generating counterarguments on claimed attributes (31) and strengthens support for the argument (16). Moreover, some studies have suggested that two-sided messages effectively improve the perceptual source credibility (32) and the perceptual trustworthiness of the advertisement (33), which further lead to positive purchase intentions (33). By contrast, other researchers have proposed different viewpoints; for example, Belch (34) and Swanson (24) have reported that purchase intentions between one- and two-sided message appeals are similar. Therefore, this study explored whether, and under what conditions, one- or two-sided messages in product claims increase the persuasiveness of communication and enhance evaluation of healthy food products of consumers.

The HSM was proposed by Chaiken (20) and has been investigated in many studies (16, 21). Chaiken (20) and Chaiken et al. (35) have indicated that the HSM involves two types of processing, a heuristic processing mode and a systematic processing mode depending on motivation and ability of perceiver. In the systematic processing mode, a comprehensive, analytical orientation, in which perceivers access, scrutinize, and integrate all the useful information, is required to make a judgment with respect to its relevance to the judgment task (36). By contrast, in the heuristic processing mode, learnt knowledge structures in the form of simple decision rules or cognition heuristics are used to make a judgment. The heuristic mode is more economical and restricted than the systematic mode. Specifically, the HSM can explain how people concurrently engage in both the systematic and heuristic modes. In other words, individuals can adopt systematic processing to obtain evidence and simultaneously adopt heuristic processing for obtaining other information in the HSM (37).

Chang (38) explained that people incorporate a systematic mode when they are not confident about judgments obtained from the heuristic mode or to maximize confidence in their judgment. A one-sided message in claims only provides supporting or favorable information of a particular product, which has been considered weak information (39). In this case, a one-sided message in claims fails to pass the “sufficiency threshold” for individuals to engage in a systematic mode of information processing because of the lack of sufficient information. By contrast, a two-sided message in claims includes evidence and arguments for both sides of a controversial issue and offers sufficient cognitive resource. In this case, individuals are inclined to systematically inspect a two-sided message. The negative information in a two-sided message may inhibit defense and strengthen supporting arguments (32, 40). Therefore, in product claims, a two-sided message is predicted to be more persuasive than a one-sided message. In other words, in product claims, individuals evaluate a two-sided message more favorably than a one-sided message.

H1: Individuals evaluate healthy food products with a two-sided message in claims more favorably than those with a one-sided message in claims.

### Effects of Mood States on Message Sidedness

The mood state significantly affects information-processing capability of individuals, i.e., people in a positive mood are inclined to process information heuristically, whereas people in a negative mood are inclined to process information systematically. For example, studies have found that people in a positive mood were less convinced by strong arguments than those in a neutral or mildly negative mood (41, 42). Similarly, Huber et al. (43) demonstrated that people in a positive mood tended to use simple information processing methods, whereas people in a negative mood employed more intense and analytic information processing methods. Evidence shows that people in a positive mood are more likely to have ready access to positive material in the memory (44) than those in a negative mood, resulting in differences in the perceived pattern and degree of relatedness among stimulus items (45).

This study analyzed how people with different mood states respond to different message sidedness in claims. This study hypothesized that a two-sided message has a greater persuasive impact than a one-sided message on people in a negative mood. According to the HSM and literature on mood states, people in a negative mood are more likely to be persuaded by strong arguments. Moreover, they are inclined to devote more time and effort to processing information through rigorous, analytical, and systematic thinking. A two-sided message provides sufficient evidence and arguments to meet the demands of individuals in a negative mood and these individuals are more likely to evaluate a two-sided message more favorably. By contrast, people in a positive mood have a more heuristic and simpler thinking style and are less likely to be convinced by strong arguments. A one-sided message appears more appealing to people in a positive mood because it offers only simple supporting arguments.

H2: An interaction between consumer mood states and message sidedness in product claims occurs when people evaluate healthy food products. Individuals in a positive mood evaluate healthy food products more favorably when a one-sided message is used, whereas individuals in a negative mood evaluate healthy food products more favorably when a two-sided message is used.

### Effects of Product Involvement on Message Sidedness

Product involvement is created by the personal significance that an individual ascribes to the features of the object (message, situation, and product). Antil (46) indicated that involvement levels may be different for different people in relation to the same object due to differences in factors such as personality, previous experience, and sociodemographic status of consumers because involvement depends on interpretation rather than the stimulus itself. Bell and Marshall (27) defined product involvement as the level of importance of a product in life of an individual. Consumers with high product involvement invest more time and effort on a specific product before making their purchase decision than those with low product involvement (47). By contrast, a low-involvement product is less crucial and relevant to needs and beliefs of consumers; therefore, consumers do not spend much time or effort to make purchase decisions (27). Involvement may influence brand loyalty, responses to product information and advertising, and even purchase decisions (27, 28, 48–53). Zaichkowsky (53) developed the personal involvement inventory to measure consumer involvement with a food product; it provides a simple measurement and an effective method of consumer segmentation (54).

This study investigated the role of product involvement in the relationship between message sidedness and healthy food product evaluation of consumers. People with high involvement in healthy food products are more likely to systematically scrutinize information and invest more time and effort in product evaluation. A two-sided message, which contains strong arguments that require a considerable amount of cognitive resources for scrutiny, is consistent with the requirements of people with high involvement. Therefore, a two-sided message is perceived as more persuasive than a one-sided message. By contrast, people with low involvement in healthy food products are less likely to invest much time or effort in product evaluation. They intrinsically lack the motivation to scrutinize information because these products are irrelevant to their personal values and interests and their evaluations of a message are probably based on simple inferences. Considering the HSM, a one-sided message incorporating supporting arguments only requires a small amount of cognitive resources for scrutiny and can be more heuristically processed than a two-sided message. Hence, people with low involvement in healthy food products are likely to evaluate a one-sided message more favorably than a two-sided message.

H3: An interaction between consumer product involvement and message sidedness in claims occurs when people evaluate healthy food products. Individuals with low involvement evaluate healthy food products more favorably when a one-sided message is used, whereas individuals with high involvement evaluate healthy food products more favorably when a two-sided message is used.

### Effects of Self-Rated Health Status on Message Sidedness

Perceptions of their own health of individuals have broadly been investigated during the last two decades. The concept of self-rated health status focuses on the assessment of personal health or health-related quality of life and personal well-being. It is a subjective measure that can be calculated at an individual level (55, 56). It presents an indication of how people perceive their health status. If individuals feel good, it indicates that their health status is either perfect or good; however, health of an individual is either average or bad if they feel bad. Self-rated health status plays a vital role in general health and quality of life (57). Self-rated health has been proposed to be not only a valid and reliable measure of health and well-being of population, but also, according to most studies, a strong predictive indicator of morbidity, mortality, and government health service utilization (58, 59).

Consumers perceive health claims differently after a change in their health status. Self-rated health condition of individuals increases their awareness and involvement with respect to food products and, hence, influences their receptiveness to relevant information. For example, individuals who feel bad about their health condition may be more concerned about health claims than those who feel good. In general, individuals with a bad health status and who are more involved are more motivated to pay attention to messages and invest more cognitive efforts in processing the message (60). This study investigated how people with different self-rated health conditions respond to different message sidedness in claims. A two-sided message is predicted to have a greater persuasive impact than a one-sided message on people with a poor self-rated health status. Considering the HSM, people process information through either the systematic or heuristic mode, depending mainly on their motivation and ability. People with poor self-rated health status are motivated to spend more cognitive resources to process the health message, which require sufficient evidence and arguments. A two-sided message in claims, which includes both the favorable and unfavorable evidence and arguments, is consistent with the requirements of people with a poor self-rated health status. In this regard, a two-sided message in claims is more likely to be evaluated more favorably than a one-sided message. By contrast, people with a good self-rated health status lack motivation to scrutinize health messages and, hence, may use simple decision-making rules instead of more demanding cognitive efforts. A one-sided message in claims, which only contains positive information and supporting arguments, appears more appealing to these types of people. In this case, a one-sided message in claims is more likely to be evaluated more favorably than a two-sided message.

H4: An interaction between self-rated health status of consumers and message sidedness in claims occurs when people evaluate healthy food products. Individuals with a good self-rated health status evaluate healthy food products more favorably when a one-sided message is used, whereas individuals with a poor self-rated health status evaluate healthy food products more favorably when a two-sided message is used.

## Research Method

### Study 1

Study 1 tested whether healthy food product evaluation of people is influenced by different message sidedness, i.e., in claims, people respond more favorably to a two-sided message than a one-sided message.

#### Method

##### Participants, Experimental Design

The participants were 60 university students (43.3% male) at a large national university in Taiwan. They were randomly assigned to one of the two experimental conditions (one- and two-sided messages).

##### Procedure

The participants were provided a booklet containing all the study materials and instruction. They were then presented with orange juice advertisement. To avoid the effect of preexisting brand preferences or prejudices, a fictitious brand name was used in the adopted questionnaire. In a one-sided message condition, the advertisement stated, “Sunplus is enriched with calcium, vitamins, and fibers. It may increase your energy level.” However, a two-sided message condition stated, “Although not very tasty, Sunplus is enriched with calcium, vitamins, and fibers. It may increase your energy level.” Then, the participants were asked to evaluate the product.

##### Dependent Measures

The dependent variables of this study were adapted from previous study and included four items whereby they capture the major attitudinal components: affect, behavioral, and cognitive (61) and were consistent with past literatures (8). Dependent measures were identical in the four studies. Participants were asked to rate the extent to which they found the product concept attractive, convincing, and credible. All the three items were assessed on a 7-point scale with endpoints labeled “absolutely not attractive/convincing/credible” and “absolutely attractive/convincing/credible.” Similarly, one item assessed intention of participant to buy the product through the question “Can you imagine yourself buying this product?” to be answered on a 7-point scale with endpoints labeled “1 = absolutely not” to “7 = absolutely.”

### Hypothesis Testing and Results

The results of the multivariate ANOVA (MANOVA) indicated that the main effect of message sidedness (Wilks' λ = 0.858, *p* < 0.05) was significant.

#### Effect of Message Sidedness on Food Product Evaluation

The results of the ANOVA analysis indicated that message sidedness had a significant main effect on product evaluation [*F*_(1,58)_ = 9.563, *p* < 0.01] and purchase intention [*F*_(1,58)_ = 6.411, *p* < 0.05]. The results revealed that a two-sided message (Mpe = 3.96, Mpi = 3.80) was associated with more favorable product evaluation and purchase intention than a one-sided message (Mpe = 3.09, Mpi = 3.03). The findings of study 1 supported H1.

### Study 2

This study tested the following hypothesis: individuals in a positive mood evaluate healthy food products more favorably when a one-sided message is used, whereas individuals in a negative mood evaluate healthy food products more favorably when a two-sided message is used.

#### Method

##### Participants, Experimental Design, and Procedure

The participants were 123 university students (45.2% male) at a large national university in Taiwan. They were randomly assigned to one of the four experimental conditions. This study used a 2 (message sidedness: one-sided vs. two-sided) × 2 (mood state: happy vs. sad) experimental design. Half of the participants were induced to feel a happy emotion, whereas the remaining participants were induced to feel a sad emotion. This study adopted a two-factor, two-level, and between-subjects design. Study 2 followed the same experimental procedure that used in study 1.

##### Mood Manipulation

Films were used to induce desired mood states, as it has been used successfully in previous study (15, 62). Participants in a sad mood condition were asked to view a segment of the film Ordinary People and those in a happy mood condition were asked to view a segment of the film Pretty Woman. Participants were instructed to watch the film, as if they were watching a video at home. After participants had finished watching the films, they were asked to complete an emotion measure.

##### Manipulation Check

Mood states were measured using the following three items: “How do you feel about the film?,” “How happy do you feel right now?,” and “Currently, I am in a good mood.” Each participant rated their current emotions. The first two scales were described with endpoints with 1 = “extremely sad” to 7 = “extremely happy” and the third scale was described with an endpoint with 1 = “strongly disagree” to 7 = “strongly agree.” Ratings of participants on the three mood items were averaged to obtain a single index and the Cronbach's alpha coefficient indicated favorable reliability (α = 0.934). Ratings of participants in the happy mood condition (*M* = 5.27) indicated that they were happier than those in the sad mood condition (*M* = 2.69). This difference was highly significant [*t*_(1,121)_ = −15.877, *p* < 0.01]. The findings confirmed the successful manipulation of moods of participants.

### Hypothesis Testing Results

Data from the MANOVA revealed an interaction between mood states and message sidedness when participants rated their product evaluation and purchase intention (Wilks' λ = 0.893, *p* < 0.01). The findings of study 2 supported H2.

#### Effects of Mood States on Message Sidedness and Food Product Evaluation

(1) Product evaluation

This study tested our predictions using the ANOVA, with product evaluation and purchase intention as dependent variables and mood states and message sidedness as independent variables. Data analysis revealed an interaction between mood states and message sidedness in product evaluation [*F*_(1,119)_ = 14.001, *p* < 0.01]. Participants in a sad mood condition rated a two-sided message (*M* = 4.24) as more attractive, convincing, and credible than a one-sided message (*M* = 3.39). However, participants in a happy mood condition rated a one-sided message (*M* = 4.06) as more attractive, convincing, and credible than a two-sided message (*M* = 3.34).

(2) Purchase Intention

**Figure 1:**
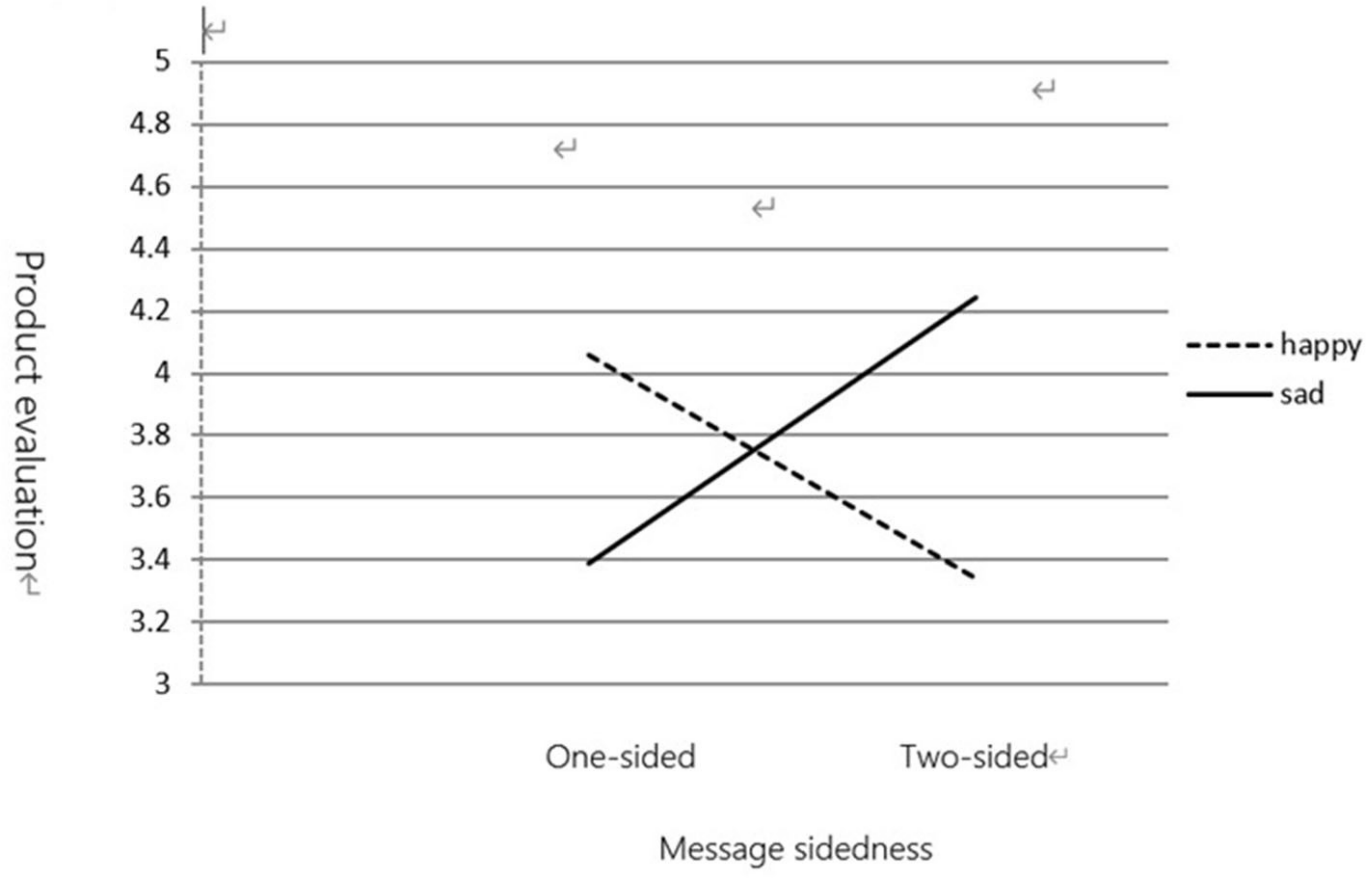
The moderating effect of mood state on message sidedness and product evaluation.

**Figure 2:**
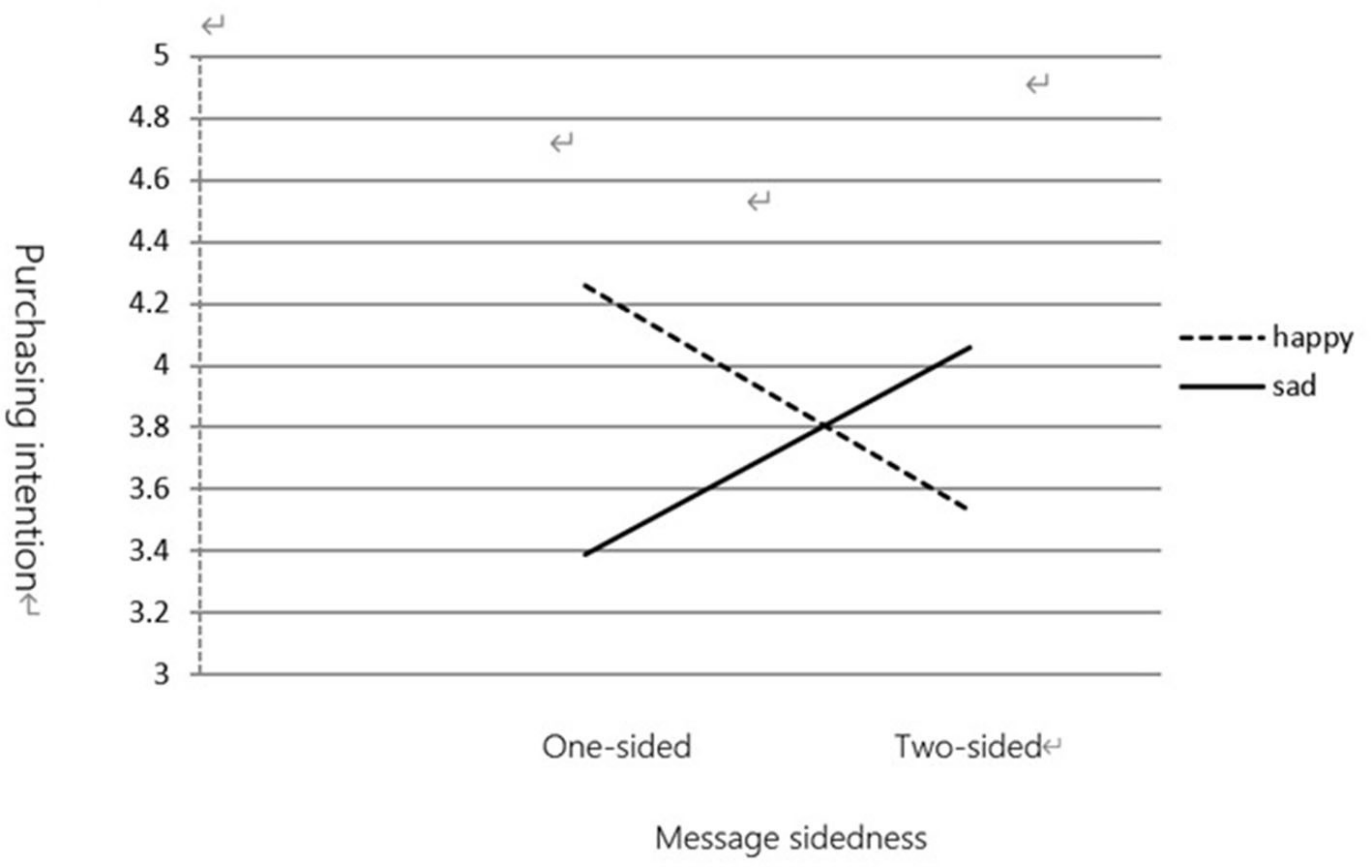
The moderating effect of mood state on message sidedness and purchasing intention.

Data analysis revealed an interaction between mood states and message sidedness [*F*_(1,119)_ = 9.145, *p* < 0.01]. As shown in Figures 1, 2, participants in a sad mood condition rated their purchase intention higher in response to a two-sided message (*M* = 4.06) than to a one-sided message (*M* = 3.39). However, participants in a happy mood condition rated their purchase intention higher in response to a one-sided message (*M* = 4.26) than to a two-sided message (*M* = 3.53).

### Study 3

This study tested the following hypothesis: individuals with low involvement evaluate healthy food products more favorably when a one-sided message is used, whereas individuals with high involvement evaluate healthy food products more favorably when a two-sided message is used.

#### Method

##### Participants, Experimental Design, and Procedure

The participants were 127 university students (42.6% males) at a large national university in Taiwan. They were randomly assigned to one of the four experimental conditions. This study used a 2 (message sidedness: one-sided vs. two-sided) × 2 (product involvement: low vs. high) experimental design. Participants were asked to complete the personal involvement inventory developed by Zaichkowsky (53). The instrument comprises the measures “To me, the juice is important/relevant means a lot to me/valuable/interesting/exciting/appealing/fascinating/needed/involving,” which is ranked from 1 (extremely disagree) to 7 (extremely agree) and a median split was used to divide participants into two groups, i.e., participants who scored above the median were categorized in the high-involvement group and those who scored below the median were categorized in the low-involvement group. Then, they were randomly assigned to one-sided or two-sided message experimental conditions. Other procedures in study 3 were identical to experimental procedures used in study 1.

### Hypothesis Testing Results

Data from the MANOVA revealed an interaction between product involvement and message sidedness when participants rated their product evaluation and purchase intention (Wilks' λ = 0.936, *p* < 0.05). The findings of study 3 supported H3.

#### Effects of Product Involvement on Message Sidedness and Food Product Evaluation

(1) Product Evaluation

This study tested our predictions using the ANOVA, with product evaluation and purchase intention as dependent variables and product involvement and message sidedness as independent variables. Data analysis revealed an interaction between product involvement and message sidedness in product evaluation [*F*_(1,123)_ = 7.619, *p* < 0.01]. Participants with low product involvement (*M* = 3.85) rated a one-sided message as more attractive, convincing, and credible than those with high product involvement (*M* = 3.39). However, participants with high product involvement (*M* = 4.20) rated a two-sided message as more attractive, convincing, and credible than those with low product involvement (*M* = 3.40).

(2) Purchase Intention

**Figure 3:**
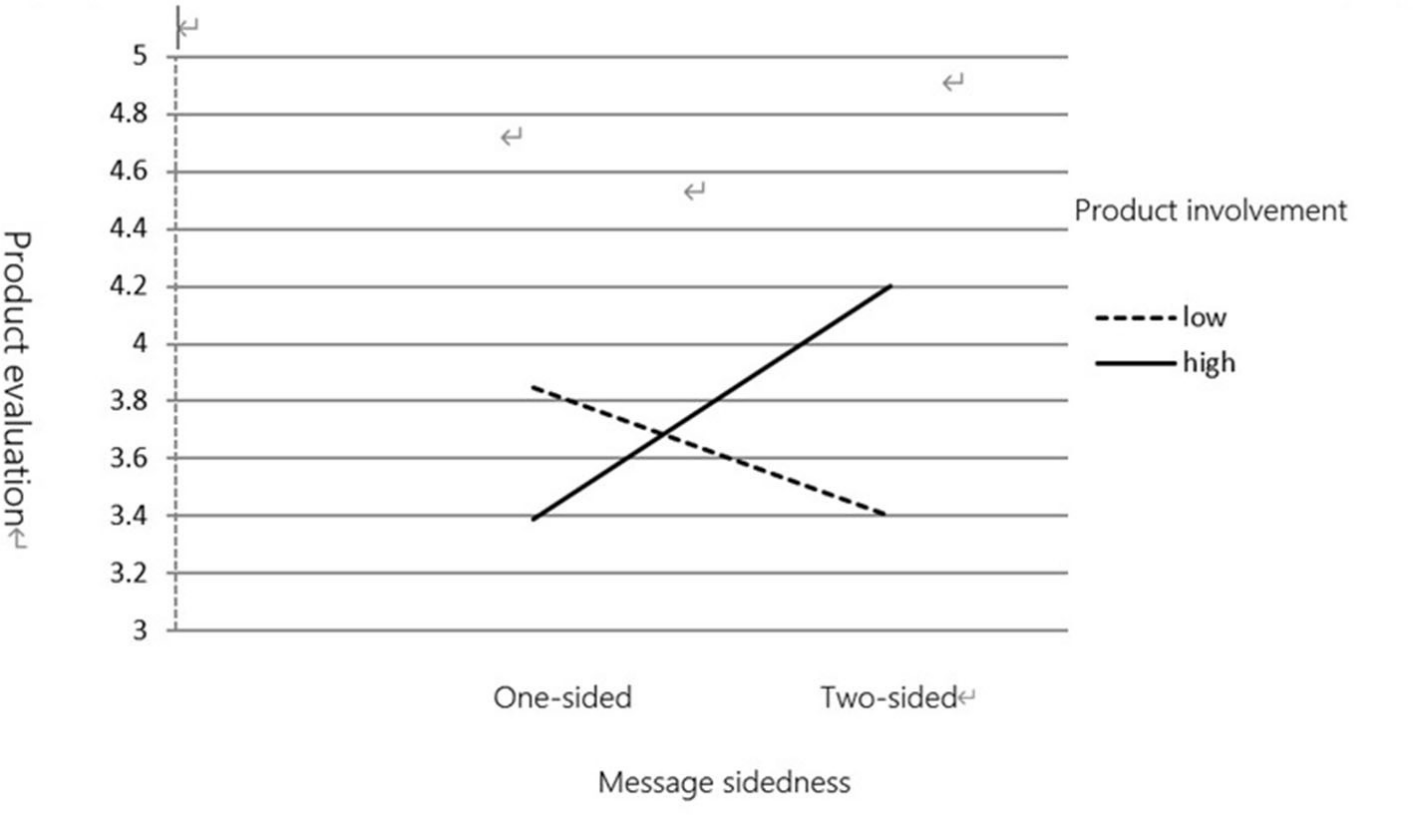
The moderatingeffect of product involvement on message sidedness and product evaluation.

**Figure 4:**
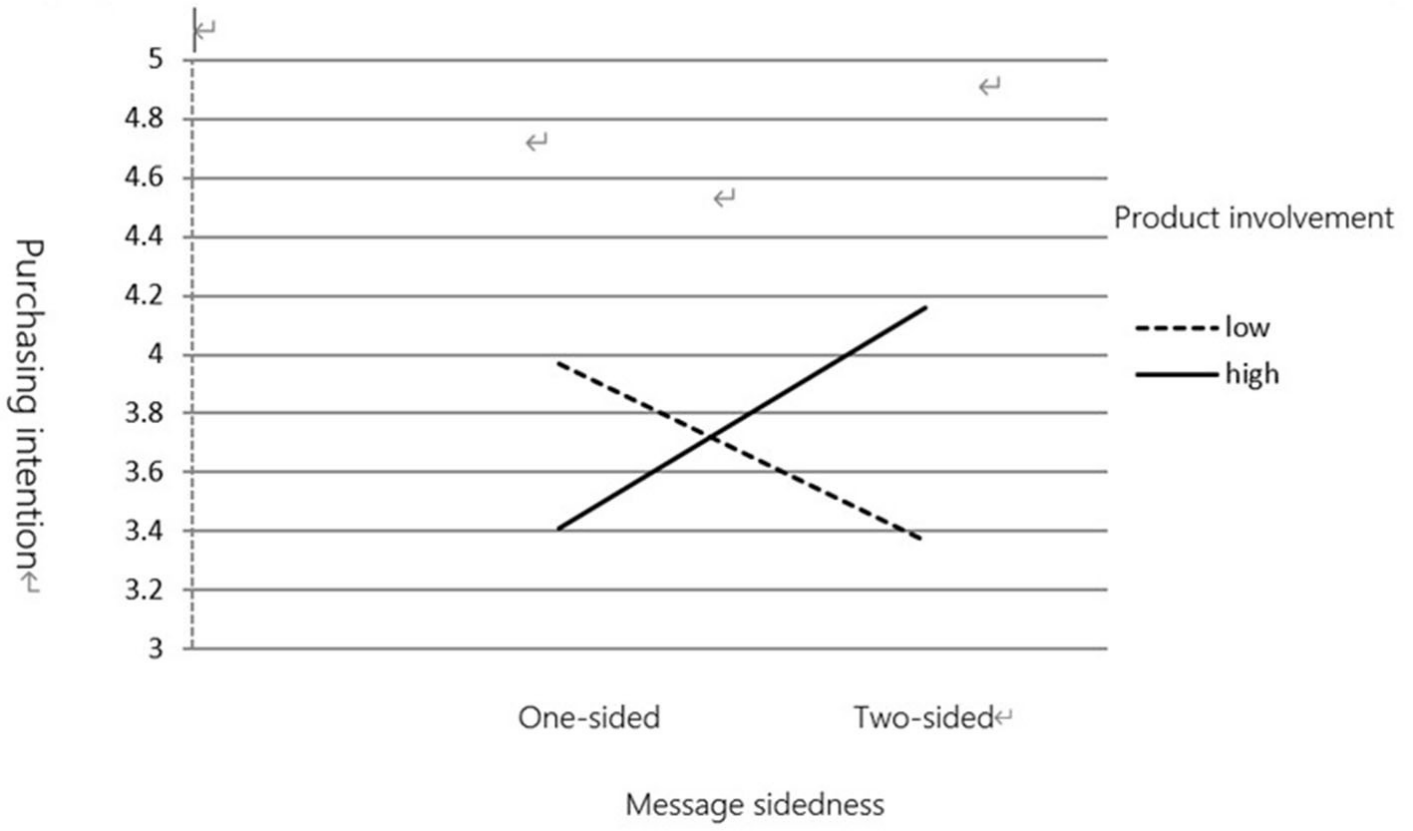
The moderating effect of product involvement on message sidedness and purchasing intention.

Data analysis revealed an interaction between product involvement and message sidedness [*F*_(1,123)_ = 7.321, *p* < 0.01]. As shown in Figures 3, 4, for a one-sided message, participants with low product involvement (*M* = 3.97) rated their purchase intention higher than those with high product involvement (*M* = 3.41). However, for a two-sided message, participants with high product involvement (*M* = 4.16) rated their purchase intention higher than those with low product involvement (*M* = 3.37).

### Study 4

This study tested the following hypothesis: individuals with a good self-rated health status evaluate healthy food products more favorably when a one-sided message is used, whereas individuals with a poor self-rated health status evaluate healthy food products more favorably when a two-sided message is used.

#### Method

##### Participants, Experimental Design, and Procedure

The participants were 120 university students (45.4% males) at a large national university in Taiwan. They were randomly assigned to one of the four experimental conditions. This study used a 2 (message sidedness: one-sided vs. two-sided) × 2 (self-rated health status: good vs. poor) experimental design. Participants were first asked to complete the self-rated health measures questionnaire. Based on the results of the self-rated health status measurement, they were then randomly assigned to one-sided or two-sided message experimental conditions. Study 4 followed the same experimental procedure used in study 1.

##### Self-Rated Health Status Measures

Self-rated health status was originally assessed using the question “How do you rate your state of health in general” offering five possible answers: (1) very good, (2) good, (3) fair, (4) bad, and (5) very bad. For the purposes of data analysis, the answers “very good” and “good” were classified together as “good” (good self-rated health status), whereas other answers were grouped together under the label “poor” (poor self-rated health status) (58).

### Hypothesis Testing Results

Data from the MANOVA revealed an interaction between self-rated health status and message sidedness when people rated their product evaluation and purchase intention (Wilks' λ = 0.936, *p* < 0.05). The findings of study 4 supported H4.

#### Effects of Self-Rated Health Measures on Message Sidedness and Food Product Evaluation

(1) Product Evaluation

This study tested our predictions using the ANOVA, with product evaluation and purchase intentions as dependent variables and self-rated health measures and message sidedness as independent variables. Data analysis revealed an interaction between self-rated health measures and message sidedness in product evaluation [*F*_(1,116)_ = 4.332, *p* < 0.05]. Participants with a good health status (*M* = 3.85) rated one-sided messages as more attractive, convincing, and credible than those with a poor health status (*M* = 3.31). However, participants with a poor health status (*M* = 3.92) rated two-sided messages as more attractive, convincing, and credible than those with a good health status (*M* = 3.42).

(2) Purchase Intention

**Figure 5:**
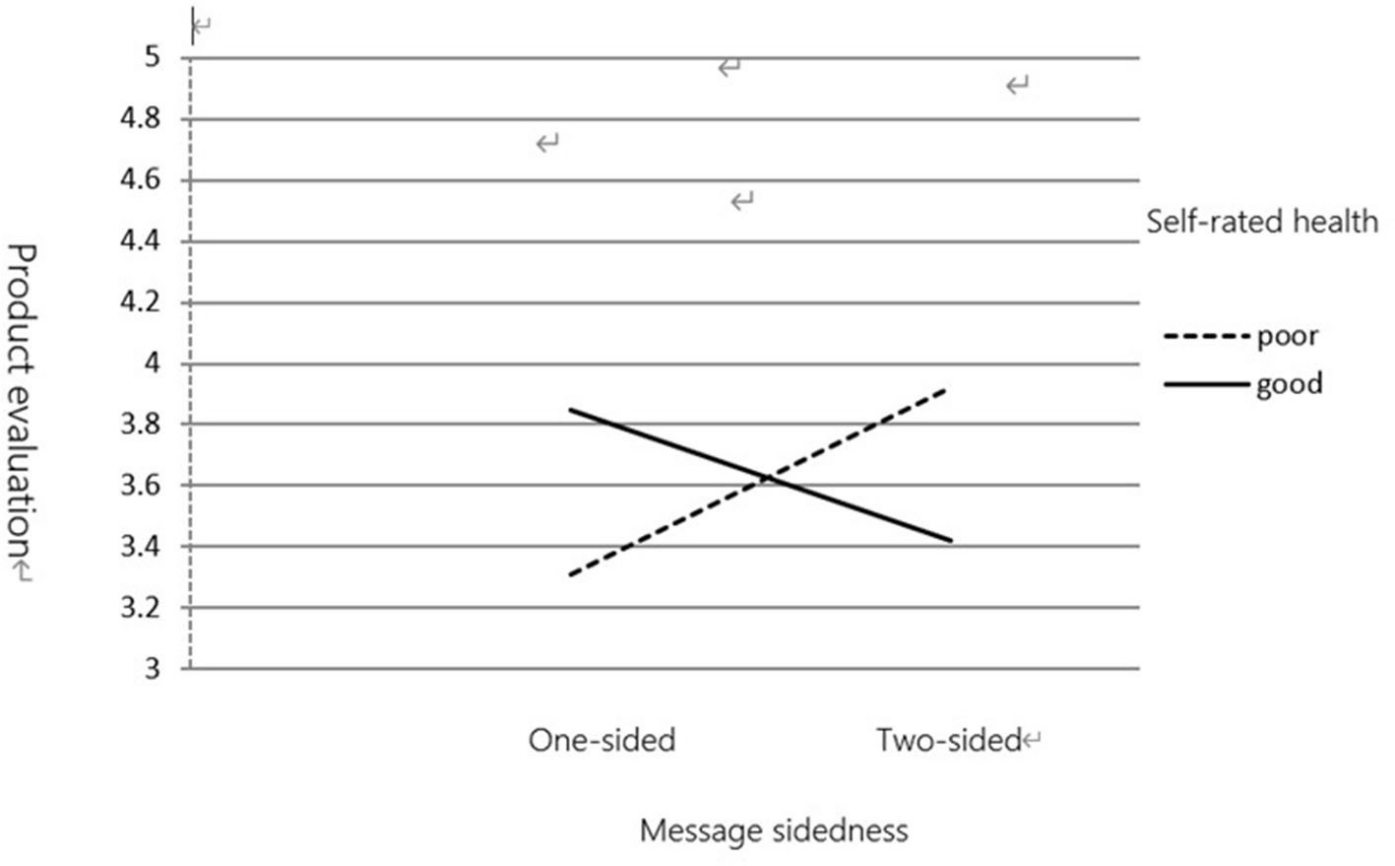
The moderating effect of self-rated health on message sidedness and product evaluation.

**Figure 6:**
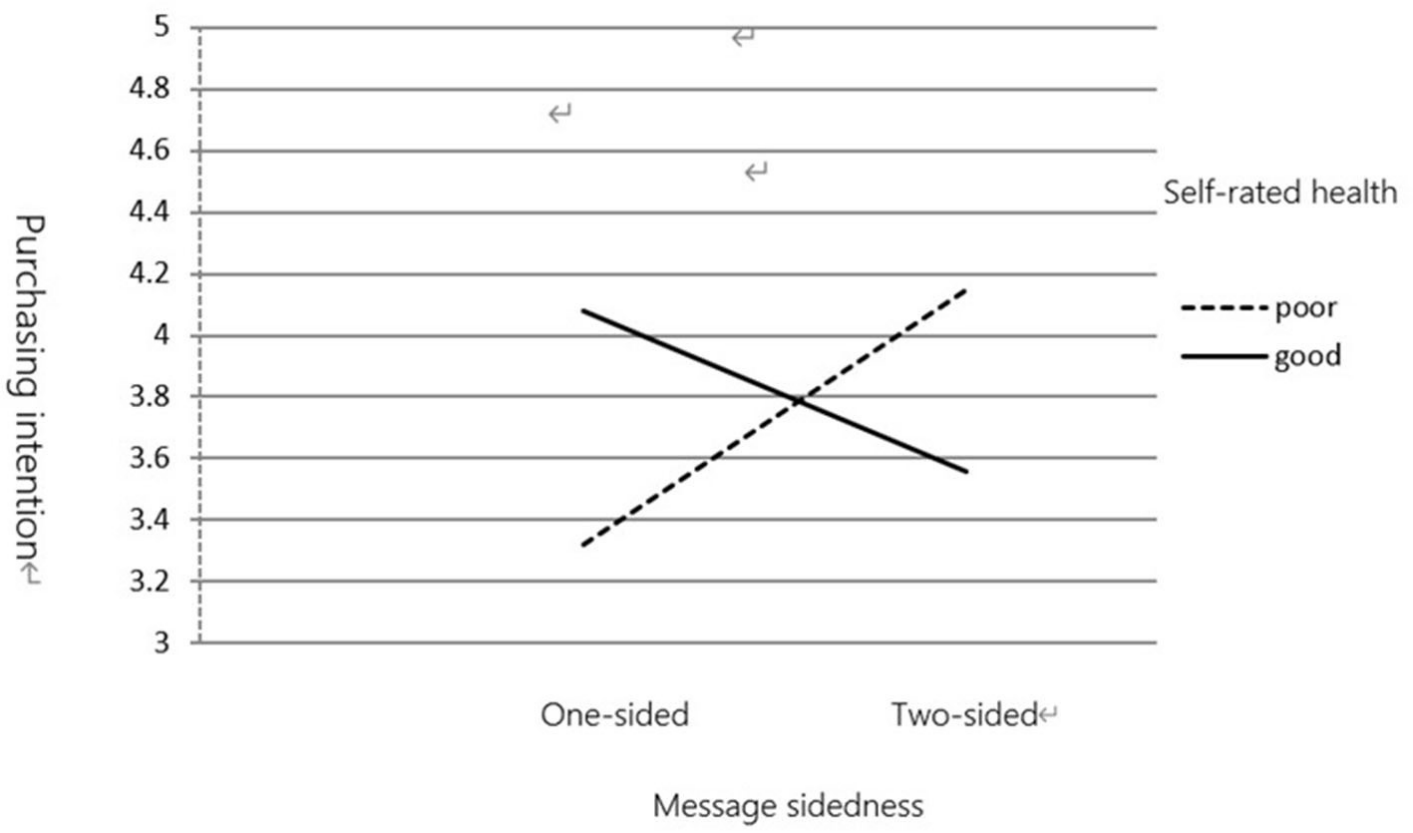
The moderating effect of self-rated health on message sidedness and purchasing intention.

Data analysis revealed an interaction between self-rated health measures and message sidedness [*F*_(1,116)_ = 7.802, *p* < 0.01]. As shown in Figures 5, 6, for one-sided messages, participants with a good health status (*M* = 4.08) rated their purchase intention higher than those with a poor health status (*M* = 3.32). However, for two-sided messages, participants with a poor health status (*M* = 4.15) rated their purchase intention higher than those with a good health status (*M* = 3.56).

## Discussion and Implications

Since most of the previous studies with respect to message sidedness mainly focus on the effect of message sidedness in advertising on behavior of consumers, this study indicates that the effect of message sidedness in claims also influence food product evaluation of consumers. Furthermore, the results of this article show that mood state, product involvement, and self-rated health of individuals moderate the relationship between message sidedness in claims and product evaluation.

Studies 1 revealed that individuals evaluate healthy food products with a two-sided message in claims more favorably than those with a one-sided message in claims. The results are consistent with the findings from Huertas and Hanna (63).

According to the HSM, a two-sided message includes evidence and arguments for both sides of a controversial issue and offers sufficient cognitive resource. The negative information in a two-sided message may inhibit defense and strengthen supporting arguments (32, 40). In this case, a two-sided message seems more persuasive than a one-sided message.

Studies 2 demonstrated that mood state moderates the relationship between message sidedness and food product evaluation of consumers. People in a positive mood tended to use simple information processing methods (64) and a one-sided message appears more appealing to people in a positive mood because it offers only simple supporting arguments. Otherwise, people in a negative mood employed more detailed attention to the communicative content of a message (65) and a two-sided message provides sufficient evidence and arguments to meet the demands of individuals in a negative mood.

Studies 3 revealed that product involvement moderates the relationship between message sidedness and food product evaluation of consumers. The results of this study are consistent with the findings from Chebat and Picard (66). People with high product involvement spend more time and effort before making their purchase decision and a two-sided message, which contains strong arguments that require a considerable number of cognitive resources for scrutiny, is consistent with the requirements of these types of people. On the other hand, people with low product involvement are less likely to invest much time or effort before making their purchase decision and a one-sided message, which incorporates supporting arguments that only require a small number of cognitive resources for scrutiny, is consistent with the requirements of these types of people.

Studies 4 demonstrated that self-rated health status moderates the relationship between message sidedness and food product evaluation of consumers. Self-rated health status has been demonstrated to be associated with health behaviors (67) and healthcare utilization (68). People with a poor self-rated health status are motivated to spend more cognitive resources to process the health message and a two-sided message, which includes both the favorable and unfavorable evidence and arguments, is consistent with the requirements of these types of people. On the contrary, people with a good self-rated health status lack motivation to scrutinize health messages, a one-sided message, which only contains positive information and supporting arguments, appear more appealing to these types of people.

This study has both the academic and practical benefits. Although empirical studies on consumer behavior have examined the effect of message sidedness (16, 31, 69), whether a one-sided or a two-sided message is more persuasive remains unclear. Moreover, most previous studies related to message sidedness have mainly focused on the effect of message sidedness in advertising on consumer behavior. How consumers respond to one-sided or two-sided messages in claims on the package of a healthy food product are unknown. Therefore, this study contributed to expand the understanding of message sidedness.

Second, this study found that the HSM proposed by Chaiken (20) explains the relationship between message sidedness in product claims and food product evaluation of consumers more effectively than the elaboration likelihood mode (ELM) (70). The HSM can employ systematic processing and heuristic processing independently and simultaneously, which makes it more suitable than ELM for evaluating the effect of message sidedness on food product evaluation of consumers.

Third, this study indicated that consumer mood states have a considerable impact on their healthy food product evaluations with respect to message sidedness. This study argued that people in a happy mood condition preferred one-sided messages in claims, whereas those in a sad mood condition preferred two-sided messages in claims, which has not been previously reported in the literature.

Fourth, this study revealed that product involvement of individuals moderates the impact of message sidedness on healthy food product evaluation. Moreover, this study demonstrated that consumers with low product involvement responded to one-sided messages in claims more favorably, whereas those with high product involvement responded to two-sided messages in claims more favorably, which has not been previously reported.

Finally, this study revealed that self-rated health status of consumers had a considerable impact on their healthy food product evaluation with respect to message sidedness. It also demonstrated that people with a good self-rated health status rated one-sided messages in claims more favorably, whereas those with a poor self-rated health status rated two-sided messages in claims more favorably.

Marketers in healthy food product companies conventionally present their health claims on products in a favorable light. However, the effectiveness of such one-sided messages on persuading consumers is unclear. The results of this study revealed different viewpoints, which may benefit marketing practitioners. First, this study suggested that a two-sided message in product claims is generally more attractive than a one-sided message. Furthermore, it indicated that a two-sided message is likely to convince consumers because it provides both the positive and negative information, which may suppress counterarguments and strengthen supporting arguments.

Second, this study suggested that the message sidedness effect can be moderated by personality traits, which implies that marketers should adopt the message sidedness strategy in combination with market segmentation. For example, people with a poor self-rated health status devote more cognitive resources and are more likely to process messages analytically; moreover, they evaluate food products more favorably when a product claim uses a two-sided message. By contrast, people with a good self-rated health status lack the motivation to scrutinize health messages and use simple decision rules; in addition, they evaluate food products more favorably when product claims use a one-sided message.

Finally, this study revealed that consumers with high product involvement respond to a two-sided message in product claims more favorably. In this study, marketers should consider marketing strategies to satisfy this consumer tendency. For example, they may emphasize the idea of buying the healthy food product as a gift for family or friends of consumer. Individuals tend to scrutinize information more carefully when making decisions to buy food products for other people. Therefore, they may evaluate food products more favorably when a product claim uses a two-sided message.

## Limitations and Future Study

One of the research limitations of this study is the use of student sample, which means the external validity of the results may be limited. Future study may use respondents with different levels of marketplace experience to determine the generalizability of the data and findings. Second, food product evaluation often takes place in the retail context, but the data of this study were gathered from laboratory-based studies and data of future study could be gathered in more realistic shopping environments.

This study has examined the moderating roles of mood state, product involvement, and self-rated health on the relationship between message sidedness in health claims and healthy food product evaluation. It mainly focused on intrinsic factors that affect evaluation of individuals and some other factors that may affect the relationship between message sidedness in health claims and healthy food product evaluation require further investigation. For example, extrinsic factors such as environmental context are worth pursuing in further study.

## Data Availability Statement

The raw data supporting the conclusions of this article will be made available by the authors, without undue reservation.

## Ethics Statement

Ethical review and approval was not required for the study on human participants in accordance with the local legislation and institutional requirements. Written informed consent from the participants was not required to participate in this study in accordance with the national legislation and the institutional requirements.

## Author Contributions

H-CL designed the work and drafted the manuscript. H-CL and S-TW collected and analyzed the data. All authors contributed to the article and approved the submitted revision.

## Funding

This study was supported by the Ministry of Science and Technology of Taiwan under grant NSC 102-2410-H-003-123.

## Conflict of Interest

The authors declare that the research was conducted in the absence of any commercial or financial relationships that could be construed as a potential conflict of interest.

## Publisher's Note

All claims expressed in this article are solely those of the authors and do not necessarily represent those of their affiliated organizations, or those of the publisher, the editors and the reviewers. Any product that may be evaluated in this article, or claim that may be made by its manufacturer, is not guaranteed or endorsed by the publisher.
